# What is ‘moral distress’ in nursing? A feminist empirical bioethics study

**DOI:** 10.1177/0969733019874492

**Published:** 2019-09-29

**Authors:** Georgina Morley, Caroline Bradbury-Jones, Jonathan Ives

**Affiliations:** University of Bristol, UK; Barts Health NHS Trust, UK; Cleveland Clinic, US; University of Birmingham, UK; University of Bristol, UK

**Keywords:** Empirical approaches, empirical bioethics, feminist ethics, moral distress, nursing practice, phenomenology, qualitative research, theory/philosophical perspectives

## Abstract

**Background:**

The phenomenon of ‘moral distress’ has continued to be a popular topic for nursing research. However, much of the scholarship has lacked conceptual clarity, and there is debate about what it means to experience moral distress. Moral distress remains an obscure concept to many clinical nurses, especially those outside of North America, and there is a lack of empirical research regarding its impact on nurses in the United Kingdom and its relevance to clinical practice.

**Research aim:**

To explore the concept of moral distress in nursing both empirically and conceptually.

**Methodology:**

Feminist interpretive phenomenology was used to explore and analyse the experiences of critical care nurses at two acute care trauma hospitals in the United Kingdom. Empirical data were analysed using Van Manen’s six steps for data analysis.

**Ethical considerations:**

The study was approved locally by the university ethics review committee and nationally by the Health Research Authority in the United Kingdom.

**Findings:**

The empirical findings suggest that psychological distress can occur in response to a variety of moral events. The moral events identified as causing psychological distress in the participants’ narratives were moral tension, moral uncertainty, moral constraint, moral conflict and moral dilemmas.

**Discussion:**

We suggest a new definition of moral distress which captures this broader range of moral events as legitimate causes of distress. We also suggest that moral distress can be sub-categroised according to the source of distress, for example, ‘moral-uncertainty distress’. We argue that this could aid in the development of interventions which attempt to address and mitigate moral distress.

**Conclusion:**

The empirical findings support the notion that narrow conceptions of moral distress fail to capture the real-life experiences of this group of critical care nurses. If these experiences resonate with other nurses and healthcare professionals, then it is likely that the definition needs to be broadened to recognise these experiences as ‘moral distress’.

## Introduction

Jameton^1^ introduced the term ‘moral distress’ (MD) to the nursing literature in the early 1980s and stated that it occurs when

one knows the right thing to do, but institutional constraints make it nearly impossible to pursue the right course of action. (p. 6)

Since then, there has been continued debate about its nature, scope and relevance with research on MD growing exponentially in recent years.^2^ Despite the popularity of MD as a research topic internationally, research and discussion regarding MD in the United Kingdom has remained limited, and as such, MD remains a relatively unknown and under-researched topic in the United Kingdom. Much of the MD research to date has focused on exploring and measuring the prevalence and deleterious effects of MD. This research has established MD as a significant problem within healthcare, thought to affect nurses and other healthcare professionals both personally and professionally.

Researchers have found that MD is associated with feelings of anger, frustration, guilt, loss of self-worth, sorrow, anxiety, misery, dread, anguish, depression and nightmares.^3,4^ Professionally, MD appears to cause clinicians to withdraw from the bedside, avoid patient contact and lose capacity to care, and ultimately, MD is reported to be an additional factor for nurses leaving or intending to leave their place of employment and the profession altogether.^5–7^ It is important to note that there is some research highlighting possibly edifying effects of being in morally distressing situations, such as increased motivation, heightening clinicians’ responses to ethical issues and providing opportunities for reflection and growth.^4,8^ The fact that MD may have some positive value does not, however, mean it is not a problem for others nor that it is a phenomenon to be ignored.

Nonetheless, when we consider the negative effects of MD and possible implications of this for a workforce that is already struggling and shrinking, then the issue of MD is significant. Recent data published by the United Kingdom’s Nursing and Midwifery Council (NMC) showed that for the first time since 2013, more nurses and midwives in the United Kingdom left the profession than joined it, and two of the most cited reasons for leaving the register were working conditions – specifically poor staffing levels and high workloads – and disillusionment with the quality of care that nurses reported feeling able to provide.^9^ The issue of retention is garnering global attention due to the economic cost of nursing turnover in both developed and developing countries,^10^ culminating in a report from the World Health Organization (WHO) highlighting the unique challenges in recruiting and retaining nurses internationally, and emphasising the vital role of the nursing workforce in improving the state of the world’s human resources for health.^11^ Given the importance of nursing to healthcare and the significant effects of MD on nurses, there is reason to research issues that affect retention and for policy makers to enact change to support the workforce. The political backdrop in the United Kingdom – notably the implications of austerity and the immanent exit of the United Kingdom from the European Union – is creating a ‘perfect storm’ as European nurses are leaving the United Kingdom because of political uncertainty and British nurses do not want to enter a profession that is increasingly stretched and disillusioned.^12,13^

To retain nurses in the profession, we need to ensure that they experience job satisfaction, and many studies suggest that nurses value environments which enable them to provide ethical, safe and high-quality patient care.^14–16^ Arguably, institutions have an obligation to support nurses who face significant moral challenges in their work and to ensure they feel supported psychologically and emotionally. The presence of MD signals ethical problems in the workplace, which can cause nurses to consider leaving that particular environment or the profession.^17,18^ However, there is conceptual confusion and debate regarding what MD is and what it means to experience MD.^19,20^ Without conceptual clarity, it is difficult to measure the prevalence or effects of, or solutions to, MD, as researchers may overlook experiences that should be regarded as MD, thus failing to accurately measure and test interventions to address it. Furthermore, there remains a lack of empirical research from a UK setting, and consequently, it is not known whether and to what extent MD affects nurses working in the National Health Service (NHS) or what potential lessons might be drawn from the UK experience for other countries and contexts.

## Research aims and objectives

This article reports some key empirical findings from the larger project, the primary aim of which was to develop a theoretically robust conceptualisation of MD that is meaningful in the context of nursing, using both theoretical and empirical methods to inform conceptual development. Prior to the empirical work reported here, a systematic review of the literature was conducted and a working definition of MD proposed:

MD is defined as:

the combination of (1) the experience of a moral event, (2) the experience of ‘psychological distress’ and (3) a direct causal relation between (1) and (2). (p. 15) (Morley et al.)^21^

In this article, we report the empirical findings that further inform and build upon this suggested working definition.

## Methodology

### Study design

The theoretical underpinning of the larger project was feminist bioethics, coined ‘feminist empirical bioethics’ following Scully,^22^ because feminist theory underpinned empirical data collection, analysis and theoretical development of MD. The exploration of women’s and oppressed individuals’ moral experiences was the starting point for our inquiry, as we sought to explore critical care nurses’ ethical experiences, uncover any oppressive practices and suggest ways to overcome them. Our commitment to ‘core feminism’: the commitment to seek and ‘eradicate traces of sexism and other oppressions wherever they may be found’ underpinned the entire project (p. 3).^23^ Scully’s^22,24^ approach to feminist empirical bioethics pays particular attention not only to gender differences but also to power differentials, marginalisation and relationships between individuals. These methodological biases and theoretical lenses enabled us to be attentive of these issues during data analysis and informed our application of the reflexive balancing method (see below) in our theoretical work.

The empirical data were gathered and analysed using feminist interpretive phenomenology, and ethical theorising undertaken using Ives^25^ method of ‘reflexive balancing’, which provided a method to synthesise our empirical data and feminist ethical considerations to draw normative conclusions about how to conceptualise MD. Reflexive balancing enables us to investigate a normative problem and generate hypotheses about it which are then tested, investigated and challenged from myriad sources (including theory and data), with the aim of either revising and specifying the hypothesis or explaining away the challenge. Our literature review^21^ generated a working hypothesis that suggested MD could feasibly be broadened, and the purpose of the empirical work presented here was to investigate to what extent UK nurses’ experiences supported or challenged that hypothesis. The aim of this article is to present the key empirical findings and consider their implications for how to conceptualise MD. A paper that presents the conceptualisation using reflexive balancing will follow this one. As such, the project is being presented in three standalone stages: (1) the literature review,^21^ (2) the empirical findings (this article) and (3) the normative argument and defence of the final conceptualisation (to be published).

### Interpretive phenomenology

The methodology used for data collection was taken from the Heideggerian tradition. Heidegger is credited with transforming Husserl’s descriptive phenomenology into an interpretive (hermeneutic) phenomenology. Husserl proposed that one could achieve understanding of phenomena by gaining a thorough description of experience, whereas Heidegger believed reaching understanding was more complex because phenomena are constantly being revealed and concealed.^26^ Heidegger rejected Husserl’s^27^ ‘eidetic reduction’ or the reduction of phenomena to their essence because, he argued, interpretation was crucial to understanding, and therefore there could not be an essence that was independent of interpretation. Researchers following the Husserlian descriptive tradition would be expected to ‘bracket’ all preconceived notions regarding the phenomenon under study to gain understanding, whereas in an interpretive project, researchers’ previous experiences and understanding are used to inform data collection and analysis. The aim is for the researcher to immerse herself in the ‘hermeneutic circle’, which, according to Koch,^28^ means moving between the singular experiences of each participant to the jointly shared experiences of all and analysing the rich data using their own interpretations. This approach was deemed the most appropriate because it allowed the first author to use their own experiences as a critical care nurse to make sense of the data.

### Feminist interpretive phenomenology

Interpretive phenomenology was combined with feminist theory to create ‘feminist interpretive phenomenology’. This might at first glance appear contradictory because phenomenology has been charged as being both essentialist and masculinist,^29^ which is not consistent with a commitment to feminism. On an essentialist reading, phenomenology is interpreted as seeking to understand the true essence of phenomena by objectifying and universalising experience. Consequently, experiences are stripped of their unique features – such as the unique perspective gender provides – and this creates the appearance of male bias at worst or gender neutrality at best. If, however, we adopt ‘progressive’ and sympathetic reading of phenomenology,^30^ then feminist theory and phenomenology can be combined (p. 6).

Heideggerian interpretive phenomenology is more readily compatible with feminist theory than descriptive phenomenology. Descriptive phenomenology – translated into a qualitative research method – seeks to provide a purely descriptive account of phenomena, and the researcher must ‘bracket’ their unique understanding of themselves, their position in the world and consequently their gender, race and social position. By contrast, Heidegger^31^ rejected the notion of ‘bracketing’ and argued that our ‘historicality’ is crucial to our understanding. The aim of interpretive phenomenology is to explore phenomena not just through lived experience but also using one’s position in the world to influence interpretation and subsequent understanding of being-in-the-world.^28^ This is more readily compatible with a feminist approach which views one’s social, cultural and political position in the world as vital for understanding. The aim of this work was to develop a unifying account of MD while recognising that it is likely there will always be experiences that fall outside of the unifying theory. Indeed, good qualitative researchers show an understanding that they are unlikely to capture the entire breadth of human experiences.

In response to the second charge of gender neutrality or worse, male bias, Fisher^29^ argues that a failure to explore female experience does not have to necessitate a male bias but rather an omission from which to begin inquiry. Indeed, prominent feminists have taken phenomenology to be a springboard from which to examine gender issues (e.g. De Beauvoir^32^ and Young^33^).

Translated into a qualitative research method, the researcher moves between their ‘historicality’ (their background understanding and preconceived notions), their experience and their interpretation of being-in-the-world to achieve understanding. As a female, cisgender critical care nurse, the lead author’s (G.M.) own experiences of gender, relationality and power, coupled with her own lived experiences of MD and working in critical care, guided probing during interviewing, data analysis and interpretation of participants’ experiences. Similarly, as described above, we entered into the empirical investigation with a working hypotheses that the concept of MD might be broadened, but not a commitment to it. Rather, we used the literature review to open up avenues of enquiry from a theoretical point of view and remained open to the idea that it might be supported or challenged by nurses’ experiences. As such, we were careful not to pre-define MD to participants but allow their own accounts of MD to emerge. To enhance trustworthiness, the research team discussed each narrative; the lead author’s interpretations were challenged, and if they could not be adequately defended were revised.

### Data collection

Purposive sampling was used to recruit nurses from critical care settings. Inclusion criteria were as follows: participants must be currently working in critical care, be registered with the NMC, English-speaking and, for one site, they needed to have 6 months or more critical care experience. This was largely due to the preferences of a gate-keeper at that site and did not seem to affect recruitment, as the majority of participants were recruited from that site.

Data were collected via face-to-face phenomenological interviews, aiming to capture participant experiences in their own words, allowing the generation of thick descriptions of MD experiences. A topic guide (Supplementary Table 1) was used to open the interviews with broad questions, but interviews were open and non-directive so that participants could lead. Importantly, MD was not pre-defined, which allowed participants’ own understandings of MD to direct the interview. Interviews lasted 120–150 min and were audio recorded with consent.

### Ethical considerations

Ethical approval was obtained from the the University of Birmingham, reviewed by the Science, Technology, Engineering and Mathematics Ethical Review Committee (project reference: ERN_15-1168S). The Health Research Authority approved the project (IRAS reference: 197577). Recruitment began at the start of August 2016.

### Data analysis

Robertson-Malt^34^ suggests that traditional philosophical hermeneutic phenomenology rejects the existence of method, but that the pragmatics of research require there to be a recognisable and structured approach. So, although Van Manen^35^ believes the analysis of a text is an art, he recognises the requirement for a set of steps and recommendations in order to develop a rigorous, replicable study. Van Manen^35^ suggests a dynamic interplay of six activities for interpretive phenomenology, and these guided the data analysis process presented in Table 1.

**Table 1.** Data analysis process.

Steps	Van Manen’s six steps	Application
Step 1	Turning to the nature of lived experience	G.M. immersed in the data – conducting interviews, making field notes, probing accounts and reflecting upon the interviews.
Step 2	Investigating experience as we live it rather than as we conceptualise it	G.M. immersed in the data – conducting interviews, making field notes, probing accounts and reflecting upon the interviews.
Step 3	Reflecting on the essential themes which characterise the phenomenon	Key experiential structures that made up each experience were hypothesised. G.M. came away from the interviews with an initial sense of some possible themes. These initial thoughts and reflections were recorded in my reflexive research diary which acted like an audit trail of data collection and analysis. These steps can increase the transparency and trustworthiness of the project.^37^
Step 4	Describing the phenomenon through the art of writing and rewriting	G.M. wrote individual narratives for each participant to capture the key elements of their MD experiences.
Step 5	Maintaining a strong and orientated relation to the phenomenon	Once the interviews were transcribed, G.M. began analysis and coding of the text in NVivo and immersed herself in the data. G.M. read and re-read the transcripts and coded line-by-line in NVivo, highlighting key words and sentences and recalling body language and emotions expressed.
Step 6	Balancing the research context by considering parts and whole	G.M. highlighted unique individual experiences and then moved forwards and backwards between the narratives, comparing the individual experiences to create shared experiences and common themes. Identifying the commonalities, G.M. constructed sub-themes which then through shared experiences, became larger themes and eventually the whole, unifying theory and definition of MD.

MD: moral distress.

Due to the time frame, we suggested recruiting 30 participants to reach data saturation. However, as Guest et al.^36^ highlight, there is limited guidance for researchers regarding how many interviews are sufficient to reach saturation. The guidance that can be found in the literature appears to be somewhat arbitrary with varying figures suggested for different methodologies. We use the idea of theoretical data saturation similar to Guest et al.^36^ as the point at which data collection and analysis no longer produce new themes or changes to existing themes. By interview 16, only a small number of new nodes were being created, most of which seemed to be unique to the individual and did not necessitate the formation of new themes; it seemed saturation may have been reached. The lack of new themes and the reduction in the number of potential participants coming forward meant that we ended recruitment after 21 interviews.

## Findings

The 21 critical care nurses who took part had varying levels of experience and different educational backgrounds. Demographic information of the participants is presented in Table 2.

**Table 2.** Demographic information of participants.

Age range (years)	Number of participants
25–34	17
35–44	2
45–54	2
Gender	
Female	18
Male	3
Hours of employment	
Full-time	18
Part-time	3
Primary clinical area	
General/trauma ITU	15
Specialist ITU	6
Seniority level^a^	
Junior	12
Senior	9
Years in current role	
<1	3
1–3	10
3–5	6
5–10	1
10–20	1
20+	0
Years registered as a nurse	
<1	0
1–3	3
3–5	3
5–10	12
10–20	1
20+	2
Highest qualification	
Bachelor’s degree in nursing	12
Diploma in Nursing	5
Postgraduate diploma in adult nursing	3
Other	1

ITU: intensive therapy unit.

^a^ Banding is determined by the NHS Employers Agenda for Change, and those in band ≥6 are considered senior nurses.

In this section, we present our interpretations of experiences described by participants as constituting MD. In the ‘Discussion’ section, we will consider what these experiences mean for the way we understand and conceptualise MD. Participants in this study discussed feeling similar distress emotions when constrained, conflicted, uncertain and experiencing moral tension and moral dilemmas. Before detailing these moral events, we will first present the prevalent emotions described by participants.

## Psychological distress

Perhaps one reason defining MD has been problematic is because the word ‘distress’ conjures up not one singular emotion but several different emotions. We agree with Paley, reported in Morley,^38^ that emphasising the breadth of emotions is not necessarily helpful for clarifying a concept, but highlighting predominant emotions can bring clarity. In Supplementary Table 2, we present our interpretation of the most commonly expressed emotions and verbatim quotations from participants to support our interpretation.

## Moral event

Some participants shared experiences they believed were examples of MD, but on further discussion of these experiences, it transpired that some were absent any moral event or ethical catalyst that caused their feelings of distress. The reflexive balancing method demands that we use this finding to challenge our prior conceptualisation that MD must be tied to a moral event of some kind, but in considering this challenge, we reasoned that MD minus a clear moral catalyst must simply be ‘distress’, and on further examination of the data, this was reflected in participant’s experience.

In this section, we present our interpretations of the kinds of moral events (constraint, tension, conflict, dilemma, and uncertainty) that participants described as causing the umbrella emotions of ‘distress’ (anger, frustration, guilt, regret, sadness and feeling torn). In the discussion, we consider whether these data support or challenge the broader conceptualisation of MD outlined in our working hypothesis.

### Moral constraint

Inability to carry out what participants described as the right thing to do was a common cause of distress, consistent with ‘constraint’ that Jameton^1^ highlighted when he first developed the term MD. When describing constraint events, participants primarily seemed to express anger, frustration, powerlessness and guilt. In the following quotation, junior nurse Joyce (all names are pseudonyms) articulates anger and frustration about a patient she believed should not have had a tracheostomy inserted. She thought that the consultant had allowed the family to make this decision to avoid engaging in a moral conflict. Joyce was unusual among this sample because unlike other junior nurses she articulated confidence in many of her beliefs and here describes how the patient should not have had a tracheostomy inserted:

I don’t think he should ever have been trach’ed [had a tracheostomy inserted] but that’s what the family wanted so that’s moral distress. It’s like the consultant has tried to get through to them but at the end of the day they didn’t say we’re going to stop things, we’re going to withdraw, but sometimes you do see that happen, sometimes the consultants do take that stand and that stand is needed but for whatever reason they decided not to take that approach this time around and now he’s just with us waiting for a bed, he’ll never go home. (Joyce)

The association of anger with injustice and moral wrongdoing is also suggested by Molewijk et al.^39^ In a paper exploring the utility of emotions within moral case deliberation, they found that anger was often associated with judgements about fairness, justice and respect. This is consistent with the work of moral psychologist Haidt^40^ who suggests that anger is a moral emotion often associated with goal blockage, frustration, betrayal and injustice. Similarly, participants in this study described feeling angry when they thought they knew the right thing but were constrained (goal blockage) and consequently felt implicated in committing a moral wrong because of their reduced agency (injustice), thus mirroring Molewijk et al.’s^39^ and Haidt’s^40^ work.

We avoid using the term ‘moral judgement’ or referring to ‘knowledge’ of the right thing because although some participants seemed to articulate certainty in their moral beliefs, other participants did not seem certain or were reluctant to express certainty. Instead, they described feeling constrained while also ‘feeling like knowing’ or ‘thinking’. This suggests that Jameton’s original stipulation that nurses only experience MD when they ‘know’ the right thing but are constrained should be understood in a weak sense. Indeed, in the next quotation, Elizabeth begins by articulating the feeling that she is not doing the right thing, and by the end of the quotation, she is doubting everything she has just said, stating, ‘we don’t want to write somebody off’:

…there’s the other side to it where you just feel like you’re not doing the right thing with those sorts of patients and I think that comes through when they are maybe older or have had really traumatic injuries…where beyond…you are beyond any doubt that this is going to turn out very poorly.…or when you get patients who have been maybe in care homes with eye tracking and really bad cognitive damage since having out of hospital cardiac arrests and you’re like I’ve seen this ten years on I know like…you really start to question whether you’re complicit in someone else’s suffering. And I do think that as a nurse sometimes you’re not really that able to like, the doctors are making a lot of clinical decisions and you’re not really able to be like, why are we doing this? What are we trying to achieve? Like are we – are we trying to achieve quantity or quality here because I think it’s quite obvious that they’re not going to achieve good quality of life but then that’s not your decision to make and that ends up being something that you do go home and just think about and think about and think about because we don’t want to write somebody off. At the same you don’t, like you feel quite cruel, maybe cruel isn’t the right word but you know you feel complicit in – in just extending suffering. (Elizabeth)

The constraints participants described were various and complex and, as Epstein and Hamric^41^ have suggested, they seemed both internal and external. However, it was often difficult to determine which constraints were internal (self-imposed) and which were external (imposed by others). For example, when participants described feeling disempowered and silenced, this could easily be attributed to their perception of their environment. However, it could also be due to working in an environment or team which really does discourage nurses from speaking up. It seems likely, therefore, that internal and external constraints are interrelated.

### Moral tension

What we have called ‘moral tension’ often seemed to be a precursor to moral conflict (see below). Participants described an awareness of moral issues and articulated a general feeling about the right thing to do but did not describe sharing their opinions with the rest of the healthcare team or trying to bring about change. In the next quotation, Amelia expresses anger and frustration as she describes feeling as if she was living in a ‘nightmare’ and a ‘horror film’, akin to being in a ‘weird dystopian’ world where patients are tortured instead of cared for. Amelia complains, legitimately, that the team was not available to speak with the family, and as a consequence, what she perceived to be futile care had to be continued. Although Amelia articulated these emotions during the interview, she did not describe sharing them with the team or acting on them. ‘Moral tension’ captures this internal struggle, where one perceives a wrong but does not articulate it openly or act on it:

‘Jesus Christ’, this just feel like a nightmare, like a horror film, like a weird dystopian thing…like we keep them alive even though they are dead! Why!? And it’s like decisions aren’t being made fast enough and they are not being made like in time, so the ward round will come around at 4pm and you’re like, well the family have already been in, they’ve gone home now so you can’t have the discussion you wanted to have with them. So, what’s going to happen now…what’s the plan? ‘Oh well we are going to wait for haematology to come in tomorrow…’. And you’re like oh great, another day of this. And it just feels cruel and it feels like its torture for this person. (Amelia)

Junior participants most often discussed feeling unprepared to deal with morally challenging situations, often describing themselves as ‘just’ nurses and believing it was not their place to question or contribute to decision-making. This lack of confidence seemed to result in moral tension because even when they felt they knew what was right, they did not share their views or act on them, and instead struggled internally. Junior participants seemed to refrain from acting on the tension and trying to bring about change by challenging the views and practices of others (what we have termed ‘moral conflict’ – see below) because they lacked the confidence to engage in ethical discussion or debate. In a review of the literature exploring nurses ethical reasoning, Goethals et al.^42^ found that often nurses had a tendency to conform to the decisions of others when faced with difficulties in ethical decision-making. Participants in our study also described doing this when they did not feel able to formulate or share their moral beliefs.

In the next quotation, junior nurse Elizabeth expresses frustration discussing her perceived lack of ethics education and knowledge, which caused her to feel unprepared to engage in ethical discussion and decision-making:

I have not had a course in ethics, I did not study philosophy I’m not sure if I’m equipped. And I think that’s really scary and there’s quite a lot of distress that comes out of that, it’s like I’m not sure if I’m really equipped to make these decisions or be part of the team that makes these decisions yet here. I am at the age of [early twenties] watching or helping someone to die and that – that’s your job and that’s what you do and it does seem very odd. I think that’s, I’ve kind of wondered that a lot in my career and just like I am, I – I’m you know that whole thing with nurses like you’re an angel it’s like no I’m not…I do not feel like I’ve got the sufficient like moral muscle to really like thrash this decision out like all the time. (Elizabeth)

### Moral conflict

In contrast to junior nurses’ reluctance to engage in moral conflict, senior nurses Rachel and Phoebe described openly challenging doctors’ decisions and engaging in ‘conflict’. In the following quotations, they both suggest that they irritate the medical team because they are constantly questioning their decisions. However, they also highlight their belief that such decisions remain out of their control:

I know that I drive the doctors mad, I’m always like, ‘but why, but why?’ and then once I get my answer I’m okay I kind of see where you’re coming from or I will say ‘I don’t agree with you, I don’t agree with that, but we’ll go with this plan but I want you to know that as a nurse I don’t agree with it and I think the bedside nurses feel the same’. (Phoebe)

--

…and there’s me rattling in there like an annoying fly. They just want to bat me away, they don’t want to deal with it because I’m just another problem for them to resolve and it’s a big thing because then they’ve got to challenge the burns doctors and there’s a whole big team of them and they think they’re right and I think I’m right but even though I’m voicing my opinions to everybody it’s not my decision is it. (Rachel)

Shared decision-making involving all members of the multi-disciplinary team is the ideal standard by which treatment decisions should be made in the NHS. However, because the responsibility for decisions ultimately lies with the doctor, they make the final decisions, which means even when nurses (or other members of the team) disagree, their opinion can be overridden, leaving them feeling ignored and disregarded.

Powerlessness further complicated participants’ experiences of moral conflicts. Due to participants’ position within the decision-making hierarchy, they were almost always constrained during a moral conflict. This might explain why many researchers accept the standard, narrow definition of MD, which suggests that moral constraint is a necessary part of MD,^43–45^ despite arguments that MD occurs due to a broader range of experiences, such as during moral conflicts^19^ and moral uncertainty.^46^

In contrast to moral tension, which is internal, moral conflict is external and involves voicing and acting on ethical concerns in a way that challenges others. Participants described feeling angry and frustrated because they could not carry out what they believed to be right and engaged in conflict to try to bring about change. Participants described engaging in moral conflicts with nursing colleagues, patients, families and most frequently with medics. In the following quotation, it is again senior nurse Rachel who discusses the frequent conflicts she engaged in with doctors regarding end-of-life care, as she describes her questioning of their rationale for treatment decisions. Rachel appeared to be frustrated and angry by what she perceived as their lack of forethought:

…with lots of situations there are patients that you just think, what are we doing? what are we doing; we’re torturing this poor patient, it’s time to stop and let them die and I have had that conversation so many times with the doctors to say, ‘what’s your plan; you’re really going to do that to them? You can’t, they’ve been through…and is that going to resolve anything?’ And because it is their decision, and it depends on how involved they are or whether they’re just covering for a shift or whatever whether they’ll just say, ‘no we’ll carry on and we’ll carry on…. (Rachel)

### Moral dilemma

While moral conflicts seemed to be characterised by frustration, anger and powerlessness, moral dilemmas seemed to have more of an enduring and lasting nature, characterised by feelings of guilt and regret. In the next quotation, Isabelle describes thinking that the terminally ill patient she cared for was articulating her wish to end life-sustaining treatment. Motivated by her responsibility to act as the patient’s advocate, Isabelle discussed the patient’s wishes with the medical team, and they initiated sedative therapy. Isabelle raised the issue of palliative care with the patient’s partner, but he became angry:

I think that day I came home fuming as opposed to devastated just thinking, and guilt, feeling so guilty…I thought okay that just happened, that was just intense, must have been a tiring day and you know I just moved on and I think it was afterwards that I realised, not pinning it all to that event but I think it really, it just, I don’t know I felt like I left a part of me in that side room that day for some reason, or like it left a scar on me that I am never going to forget and I felt like it was the right thing to do but it genuinely, I don’t know it makes you think about things that you wouldn’t see otherwise…like because you spend so much time at the bedside you end up getting to know the patient more than the doctors often, or the rest of the team. And then you have to stand up for people, for patients, I find…and sometimes that is, you know you can be torn thinking you know, is this right? Have I gone too far? Am I just going crazy? Am I just tired? And not just for life or death situations but just in general, am I pushing it too far like standing even for them thinking I know this isn’t all that you want but I am going to do it because I know this is what you need right now, and even then you can feel like you know, am I doing the right thing? Is this really the right thing to do? And it brings some sort of tension that you can’t quite explain or you don’t really think about it you just come in and then you leave work but I genuinely think it leaves a mark on you in some way so it does affect you in some ways that you couldn’t quite explain…did I misunderstand, did we all misunderstand this? What is the right thing to do? What’s my job? Is it to look after her or the family, or both? And in that case what do I do, when both interests seem to be different? Yeah, I think that was the main thing, just and if it was the right thing why does it feel so hard and so painful, because often if you do the right thing you go home satisfied thinking I have done what I am supposed to do…That day was probably the worst day where I didn’t feel like I had done the right thing and looking back I am convinced it was the right thing, it just did not feel like that and for a long time it still didn’t feel like that. (Isabelle)

Isabelle seemed to find it difficult to settle on ‘the right thing’ as she asks multiple rhetorical questions. She suggests that this experience brought ‘some sort of tension that you can’t quite explain’; even though she carried out the action she believed was morally right (by raising the issue of palliative care with the patient’s partner), she still describes experiencing pain and guilt. Isabelle seemed to be tormented as she describes the feelings of regret, guilt and loss that she experienced, stating ‘if it was the right thing why does it feel so hard and so painful’ and feeling as if ‘I left a part of me in that side room that day…or like it left a scar on me that I am never going to forget’. We suggest that this feeling, as Williams^47^ and Marcus^48^ argue, is ‘moral residue’ or ‘moral remainder’ which signals that Isabelle experienced a ‘genuine’ moral dilemma, leaving Isabelle feeling uncertain and conflicted about whether she should have prioritised her obligation to the family rather than to the patient. Isabelle describes the lingering feelings of regret as she continued to wonder whether she had truly done the right thing, despite also feeling that she had.

### Moral uncertainty

Participants described feeling torn, conflicted, frustrated, guilty and upset because they experienced moral uncertainty. In the next quotation, Holly is responding to the question, ‘what do you think moral distress is?’ Many participants discussed the complexities of ethical decision-making and expressed the belief that because medical prognostication is often uncertain, this necessarily makes many moral decisions uncertain. This caused them to feel, as Holly describes, conflicted and tormented as they struggled to navigate the ethical challenges they faced:

Where you’re in torment and conflict because of the morality, the rightness or wrongness of a situation and it’s a, it’s a very visceral thing, actually, I feel it’s sort of, um, it’s an instinctive thing, it’s a physical reaction almost that gets you before the consciousness of it. (Holly)

Jameton’s^1,49^ conceptions of MD, and those subsequent to it,^41^ have built on the assertion that MD only occurs when one has made a moral judgement but is constrained. However, our data suggest that being unable to make a moral judgement is also a powerful cause of distress. In the next quotation, Phoebe suggests she feels more distressed when uncertain than constrained because the uncertainty makes her feel more powerless and unable to even articulate an opinion:

G.M.: So, in the situations where you’re not totally sure that you know the right thing to do, do you think that would still cause you moral distress, would you still feel morally distressed?

Phoebe: Yeah because I’d then probably feel guilty that I knew it wasn’t the right thing but I didn’t know what the right thing was. That would almost probably make me feel worse.

G.M.: Yeah, really?

Phoebe: Yeah because I wouldn’t be able to fight my corner. If I’m out there and I know that we’re doing the wrong thing then I can say ‘this isn’t right, what about this, what about this?’ I can verbalise what I’m thinking, what I’m feeling and then I can have feedback and I can have the doctor say well this, this and this and you can have a conversation about it, but I think those situations where it’s like this isn’t right but I don’t know why, that causes me quite a lot of distress and then I would feel almost a bit guilty because I’m like I know we’re not doing the right thing but I don’t know what the right thing is and then I feel like I’m failing a little bit because I feel I should know what the right thing is.

## Discussion

Although Jameton’s narrow conception of MD has remained prevalent within the MD literature, several authors have problematised his characterisation of not only MD but also of moral conflicts and dilemmas.^19,46^ Fourie,^19^ for example, highlighted how Jameton’s^1,49^ inconsistent use of the terms ‘moral conflict’ and ‘moral dilemma’ suggested that he accepted a ‘common-sense’ definition of moral dilemmas, on which a dilemma is a difficult moral decision, but, with careful analysis, one can identify the morally correct course of action and show that the other is not required. The consequence of this, argues Fourie,^19^ is that Jameton^1,49^ viewed dilemmas and conflicts as interchangeable, leading to the conclusion that on his account MD could be regarded as mutually exclusive to both dilemmas and conflict (thus creating an extremely narrow conception of MD). In this section, we will discuss the way in which we understood and defined conflicts and dilemmas, and highlight how participants seemed to feel distressed during both moral events.

To make sense of participants’ experiences, we drew upon the work of Tessman^50,51^ for our understanding of moral conflicts and dilemmas. Tessman^50,51^ suggests that although moral conflicts and moral dilemmas are similar, they remain distinctly different phenomena. She defines moral conflicts as

A situation in which:

1. there is a moral requirement to do A and a moral requirement to do B; and

2. one cannot do both A and B. (p. 27)^51^

As Tessman^51^ explains, in conflicts one can ‘for the purpose of determining what you ought to actually do, take one of the moral requirements to override the other one’ (p. 28). This seemed to reflect the way participants experienced moral conflicts. Participants described feeling angry and frustrated because despite openly engaging in discussion with others to advocate for their view, they could not carry out the moral requirement they believed to override the other. For example, in the quotation from Rachel in the previous section, she discusses the frustration associated with an end-of-life experience in which she believed their duty to end life-sustaining treatments to prevent suffering (A) overrode the moral requirement to sustain life at all costs (B). There was a conflict between A and B, which for the purposes of action guidance one had to decide (in this case the doctor) to override A with B.

In contrast, a moral dilemma is defined as

a situation in which there is a moral requirement to do [or to refrain from] A and a moral requirement to do [or to refrain from] B, where one cannot both do [or refrain from] A and do [or refrain from] B, and where neither moral requirement ceases to be a moral requirement just because it conflicts with another moral requirement, even if for the purpose of action-guidance it is overridden. In a dilemma, whichever action one chooses to perform [or refrain from], one violates what has become, through one’s choice, the impossible moral requirement to do [or refrain from] the other action. (p. 15)^50^

Reflecting on Isabelle’s experience, she seemed to be suggesting that by carrying out one moral requirement (to act according to the family’s wishes and their interpretation of best interests), the other moral requirement (to act in the best interests of the patient) was not successfully overridden and the patient was forced to bear a cost that no one should have to bear (continuing life-sustaining treatments despite a terminal diagnosis and therefore continued pain and suffering). This caused Isabelle to experience moral residue: the residual feelings of guilt, anger and regret that signal the violation of a non-negotiable moral requirement.^47,48^ Moral conflicts can be distinguished from dilemmas in our data because of their lasting and residual effects. When participants described moral conflicts, they described psychological distress, but they did not describe the associated residue because – applying Tessman’s definition to their accounts – the moral requirement in a moral conflict is negotiable: one moral requirement could be successfully negotiated for the other. Conversely, the moral requirement in a dilemma is non-negotiable, so although the moral agent may choose (for the purpose of action guidance) to fulfil one moral requirement instead of the other, this does not mean that it ceases to be a requirement. The residual guilt and regret signals the continued pull of the non-negotiable moral requirement.

Even if the distinctions we have drawn between dilemmas and conflicts are not fully accepted, what does seem clear is the way in which psychological distress is infused in each event. This problematises Jameton’s distinctions between MD, moral conflicts, uncertainty and dilemmas, and provides empirical support for arguments made by Fourie^19^ and Campbell et al.,^46^ who suggest our understanding of MD ought to be broadened beyond constraint. The participants in our study clearly articulated distress that was causally associated with feeling morally conflicted, uncertain and torn, which supports Fourie’s^19^ claim that

…a definition of moral distress, which makes constraint central to distress, seems to distort the reality of the situation. Whilst constraint may be present and its significance should not be under-estimated…the case does not seem to be one that is accurately portrayed as being primarily about constraint: it is not simply that other people are arbitrarily or unfairly standing in the nurse’s way but that they genuinely disagree with the nurse on a moral basis. (p. 97)

Our findings suggest that narrow ‘constraint’ definitions of MD are not consistent with the actual experiences of these critical care nurses. In light of these findings, we suggest that our preliminary definition of MD might be further specified as follows:

1. The experience of a moral event
  ‘Moral event’ could be any/combination of the following: moral tension, moral conflict, moral dilemma, moral uncertainty or moral constraint.
2. The experience of psychological distress
  The term ‘psychological distress’ is an umbrella term that captures a variety of different negative emotions that may be expressed differently by each individual but predominant emotions are anger, frustration, guilt, regret, sadness/upset, powerlessness, symptoms associated with stress and feeling torn.
3. A direct causal relation between 1 and 2.

Broadening the definition of MD so that it includes a range of moral events may increase the relevance of the concept as it captures more accurately the various ethics-related distress experiences of healthcare professionals. To further differentiate between these experiences, we can, as Fourie^19,20^ suggests, sub-categorise distress to ‘moral-constraint distress’ and ‘moral-conflict distress’, to which we would suggest adding ‘moral-uncertainty distress’, ‘moral-dilemma distress’ and ‘moral-tension distress’.

We have highlighted how our findings suggest certain emotions may be associated with particular moral events (Table 3). This may provide us with a key to understanding and addressing MD. If individuals can identify their predominant emotions, this could help them to identify the underlying moral event and possibly the best way to respond.

**Table 3.** Summary of moral events.

Moral event	Description	Predominant emotions
Constraint	The moral agent may feel/know/believe which moral requirement to fulfil (for the purpose of action guidance) but is unable to carry out their preferred moral requirement due to (perceived or real) external or internal constraint. There is no moral residue.	Anger, frustration, powerlessness and guilt – the moral agent feels/knows/believes the right thing but feels, or is actually, constrained. This may result in the perception they have committed a moral wrong.
Conflict	The moral agent may feel/know/believe (for the purpose of action guidance) which moral requirement to fulfil, substituting one for another. The moral agent may be prevented from enacting/fulfilling their moral agency because of a constraint. There is no moral residue.	Anger, frustration, powerlessness and sadness/upset – the moral agent engages in conflict but unable to fulfil their preferred moral requirement.
Tension	The moral agent may feel/know/believe (for the purpose of action guidance) which moral requirement to fulfil but is/feels constrained and therefore refrains from engaging in actual conflict. The moral agent may believe they have committed a moral wrong. There is no moral residue.	Anger, frustration, powerlessness and guilt – a precursor to conflict as the moral agent feels/knows/believes the right thing but feels, or is actually, constrained and unable to engage in moral conflict with others. This may result in the perception they have committed a moral wrong.
Dilemma	The moral agent is unable to decide/is unable to fulfil two or more non-negotiable moral requirements that cannot be substituted. There is moral residue.	Guilt, torn, frustration and sadness/upset – the moral agent may be unable to decide between two or more moral requirements; the moral agent may feel the loss of the unfulfilled moral requirement (initial emotions). Regret, guilt and sadness/upset – the moral agent may feel the loss of the unfulfilled moral requirement (residual emotions).
Uncertainty	The moral agent is uncertain/unable to decide which moral requirement to fulfil – if the moral requirement is non-negotiable then they may experience moral residue.	Torn, frustration and guilt – the moral agent feels conflicted and uncertain about the right thing to do (initial emotions). Regret and guilt – the moral agent feels uncertain about whether they prioritised the correct moral requirement (residual emotions).

For example, if the moral agent is feeling torn, this may indicate they are experiencing moral-uncertainty distress or moral-dilemma distress. One way to respond to this would be to provide the agent with moral guidance so that they can discuss their ethical concerns and identify an ethically justifiable conclusion. Similarly, if an individual is experiencing moral-constraint distress, then it may help to try and understand what the constraint is, whether there is a justification for it, and if not, identify a way to negotiate the constraint.

## Limitations

There are limitations to the sample that may affect the transferability of the findings. Although the interviews were in-depth and provided rich data, the sample size was relatively small. All participants were European and only one participant was from a minority background. The findings presented are based on our interpretation of participants’ reported experiences, and therefore if our interpretations are fallible, then so are our conclusions. We have tried to maintain trustworthiness in this process by explaining our interpretations and providing verbatim quotations to support interpretations.

In both phenomenology and feminist research, the aim is to try and build common themes through shared experiences, and this does require some generalisation. However, one must always be aware of the risk that any generalisation can contribute to the oppression of those whose experiences that do not ‘fit’.^52^

## Conclusion

We suggest that the characterisation of MD offered in this article is likely to be applicable across different practice areas and settings, but the predominant emotions may vary according to cultures and contexts. By broadening the definition of MD, we believe we have paved the way for a more inclusive conception of MD that has international relevance beyond this particular group of critical care nurses.

## Supplemental material

SUPPLEMENTARY_INFORMATION_-resubmission - What is ‘moral distress’ in nursing? A feminist empirical bioethics study SUPPLEMENTARY_INFORMATION_-resubmission for What is ‘moral distress’ in nursing? A feminist empirical bioethics study by Georgina Morley, Caroline Bradbury-Jones and Jonathan Ives in Nursing Ethics
