# Supplementary material for: What is ‘moral distress’ in nursing? A feminist empirical bioethics study
Source: Nurs Ethics. 2019 Sep 29;27(5):1297–314. doi: 10.1177/0969733019874492 (PMC7406988; doi:10.1177/0969733019874492)
Supplement: SUPPLEMENTARY_INFORMATION_-resubmission - What is ‘moral distress’ in nursing? A feminist empirical bioethics study [file SUPPLEMENTARY_INFORMATION_-resubmission.pdf]

SUPPLEMENTARY INFORMATION

Supplementary Table 1: Interview Guide

The questions and probes below are illustrative only and do not represent a script. Whilst they indicate the scope and tone of topics that may be raised, the conduct or content of the interview cannot be scripted in advance.

The overarching method for these interviews is 'Feminist Interpretive Phenomenology' and consistent with this, questions are left as open as possible so that the participant can select which experiences they want to discuss.

Interview Section	Suggested Questions
**Introduction**	- Introduce self and project.<br>- Assure confidentiality: unless any cause for concern about yours or anyone else's safety – then a senior colleague may be notified.<br>- Confirm consent, consent to interview, consent form initialed and signed with a copy to keep.<br>- Remind participant of 4-week window to withdraw and refer to designated number for withdrawal.<br>- No right or wrong answers.
**Clinical Background**	- Age<br>- Clinical area/speciality<br>- Years in current role<br>- Years registered as a nurse<br>- Education
**Broad Beginning Questions**	1. What sort of work-related issues are likely to preoccupy you after your shift has finished?<br>2. What, if anything, that is work-related, keeps you awake at night?<br>3. What kind of issues do you find challenging or distressing at work?<br>4. Does anything bother you about work?<br>5. Are there any clinical scenarios or encounters that you find challenging?<br>6. Can you describe the sorts of ethical issues/problems that you encounter at work?<br>7. Can you think of a time where you felt compromised as a nurse?<br>8. What do you think moral distress is?<br>9. Do you find moral distress to be a negative or a positive experience?

**Suggested Probes**	1. Can you provide a background of the patient, such as why they were in hospital?<br>2. Can you describe more about the experience?<br>3. Can you describe the ethical issues?<br>4. Can you describe how the experience made you feel?<br>5. Can you describe how the experience made you feel afterwards?<br>6. Can you describe any thoughts/feelings you might have had after the experience?<br>7. Do you think this experience impacted your practice in anyway?<br>8. How do you think you would respond in the future to a similar situation?<br>9. Can you provide an example?
**Suggested Follow-Up Questions**	1. Did you seek any support during, or after this experience?<br>a) If so, what kind – formal/informal?<br>b) Did you find that support helpful?<br>2. Did you receive any support during or after this experience?<br>a) If so, what kind – formal/informal?<br>b) Did you find that support helpful?<br>3. Did you find anything you did yourself to be helpful?<br>4. What support would you find helpful in the future should a similar situation occur><br>5. Did you reflect upon the experience afterwards?<br>6. Did you discuss the experience with anyone?<br>7. Did you discuss the experience with your colleagues?<br>8. How did you find the response of your colleagues during/after your experience?<br>9. Has your experience affected the way you respond to your colleagues?
**Generic Probes**	- Can you tell me a bit more about that?<br>- And then what happened?<br>- How did you feel about that?<br>- What was the most difficult/positive aspect?<br>- What was helpful?<br>- What worked well?<br>- What help would you have liked?

Supplementary Table 2: Predominant Emotions Expressed by Participants

Emotion	Participant Quotation	Interpretation
**Anger**	*"…they all f*** off and it's just me and the locum reg [a senior doctor who is temporary] who I knew was lovely and I'm like her blood pressure's just f***ing going so cranking up the norad [continuous intravenous medication to support blood pressure]… I'm like ringing the nurse in charge, he's like, erm, he's like oh, it's just your transducers like it's not just my f***ing transducers, brother [transducers must be at a certain level to accurately measure blood pressure]. So then my other colleague who is solid, I say 'Richard, I need you in here'. I knew she was going to arrest. This poor doctor was trying to get other access in where her Vascath [intravenous access through which emergency medicine can be given] was beeping and alarming, the family were probably in some kinda happy oblivion because she'd been nearly dead so many f***ing times, why would they think today was gonna be the day; they probably wouldn't think that. So, she did, she arrested and they put paddles [to provide shocks during a cardiac arrest] on and she, her skin was sliding around her body like plate tectonics and there was blood all through the bed…. Mum and dad weren't in*	Holly's narrative was littered with expletives as she describes the moment a patient suffered a cardiac arrest and died after receiving what Holly perceived to be as futile and aggressive care. Holly seems to describe feeling a sense of injustice because the patient was receiving futile care and she felt obligated to attempt resuscitation without adequate support (working only with a locum registrar and struggling to contact the charge nurse) despite her belief the patient had no chance of survival.

	there when…I don't think they got to spend time with her alive but I was heartbroken for her, for us [crying] for the fact that that situation should've never been allowed to happen. This was over two years ago and you can see the effect it's had on me. So, when my shift ended I, I was just like shit, I have to get a taxi home, I was just like shell-shocked, people are like are you alright, I was just like, no. I had like four days off as part of my rota after that. I helped, I stayed late to help lay her out 'cos that was sort of somehow sort of helped. The family were cool but obviously not. That was just the most brutal thing to have to experience." (Holly)	
**Frustration**	*"It's frustrating. It is frustrating. It's a bit de-motivating when you think how much money, time and effort is going into that patient to know that really they're not going to survive it, they're not going to get out of it …but what they learned from putting that man on ECMO was so educational for other traumas on ECMO that now we're putting more traumas on ECMO and saving more lives in that way but then for every one of him, how many have we put on that haven't survived? But then those people would have died anyway, do you know what I mean? So, is that a waste of money and resources, could somebody else have had that*	Phoebe describes the frustration associated with treating a patient on extracorpoeal membrane oxygenation (ECMO) and articulates uncertainty as she considers the benefit of using an expensive and resource-intensive intervention for a patient she believes is unlikely to benefit. Phoebe discusses weighing the level of suffering with the potential benefit of further learning. Phoebe concludes by stating that many ethical decisions in critical care are rarely clearly right or wrong, and seems frustrated by this uncertainty.

	as a resource, or, did we learn from that okay we can put this trauma on but not that trauma on? As with everything in Intensive Care, nothing is ever black and white as much as I would like it to be….” *(Phoebe)*	
**Guilt**	*"I still felt guilty because I knew she didn't want me to do it, and as I say we are taught from day one about autonomy and about capacity and consent, and I knew she had capacity and technically she was not giving me consent to suction her via her trache[ostomy] but it's that very hard grey area of best interests, you know? I'm not allowed to just allow you to plug off, so it is difficult. It's hard when you try to say right and wrong which is the difficult part of it but I knew I needed to do these things but it didn't stop me from feeling guilty about it."* *(Beth)*	Guilt seemed to be associated with moral constraint and moral uncertainty. Beth described feeling constrained because of her professional obligation to continue providing life-sustaining treatment (in this example, suctioning an artificial airway) but she also expressed uncertainty because this conflicted with her personal feeling of relational responsibility to the patient. Beth seemed to feel residual guilt because she was uncertain about whether she had acted in the patient's best interests and done the right thing which could signal this experience as one of a moral dilemma as she continued to doubt whether they had done the right thing by continuing life-sustaining treatments and invasive procedures. For both guilt and regret there seemed to be a difference between emotions felt in the moment and those that lingered. Lingering feelings of guilt and regret seemed to signal moral residue and the experience of a moral dilemma.
**Regret**	*"I don't know, the nature of the beast sometimes, is that you think like there's always an element of like, kind of, 'What more you could have done? Could I have done this better? Did I... put my opinion across enough? Did I advocate for my patient appropriately?' That kind of thing ... but it's the stuff that really stays with you and then makes you think about things and it is... I find the more kind of responsibility you have in a role, the more you start questioning maybe*	Regret seemed to suggest a feeling of loss and was often described alongside guilt. Grace suggests regret and guilt are almost inevitable, stating that it is the *"nature of the beast"*. Grace seems to suggest that truly distressing events are those that cause moral uncertainty and leave you questioning. The residual feelings of guilt and regret seemed to be associated with continued uncertainty, signaling moral residue and the experience of a moral dilemma.

	morals and stuff because when like I was kind of first starting out as a bedside nurse, you're kind of – you're learning – you're almost learning the trade, you're learning how to look after the patients appropriately and … you're doing what is told because, you know, you, you haven't got the experience and stuff and then the more experienced you get, you start thinking, you know, 'Oh well, maybe we could do this as well' and, you know, that's great and then... going into different roles, like... doing – I mean doing Outreach senior [nurses that provide assistance for deteriorating patients] was just like every single shift, it felt like you're questioning, 'Is this the right thing to be doing?' And a lot of that would be whether we should admit someone to the ITU or not but then it's very difficult to say, 'actually, we shouldn't admit them'. Like, what are the reasons?... and that's kind of where you have to delve a little bit deeper into the patients and think about the quality of life and that kind of stuff and actually what they're presenting complaint is but I found that really difficult, especially when... like again, with – there would be differences in opinions between the Outreach Nurses and the Registrars [junior doctor undertaking specialty training] …I found that role particularly difficult with,	

	with that kind of thing about doing the right thing for patients and there being a real difference in opinion." *(Grace)*	
**Sadness/ Upset**	*"I think when you're at the bedside for someone, and I guess nurses say this quite a lot. When you're the person that's there with them for twelve and a half, thirteen hours a day, it's very difficult not, and you maybe shouldn't, but it's difficult not to get wrapped up in how they feel. Visiting teams, though I'm very sure they were doing their very best for her. I don't think they kind of succumb to that the way we would because you're feeling the full force of someone's distress…all day. You're feeling the full force of their family's distress for most of the day so it's really hard to kind of take that step back and be more practical or more logical in your thinking. You know, I wasn't, I wasn't crying at the bedside, I wasn't in a state, but I felt it. And there's obviously, you know, that didn't leave the minute you walked out the door, it stayed with you, and feeling that for hours and hours on end for a day is draining! It's draining on anyone! ...yeah… it's difficult. It's one of those difficult scenarios where I don't think anyone did anything wrong but it still didn't feel right in the end."* (Beth)	Feelings of sorrow and sadness were commonly expressed emotions, and sadness seemed to be experienced primarily during moral conflicts and dilemmas. Beth describes how nurses, patients and families experience emotive situations together and because they are in close proximity for long periods of time they can form intimate relationships. When there is an emotional connection this seemed to make managing moral conflicts and dilemmas more difficult. For example, Beth describes how she finds it difficult to detach herself and adopt the *"practical"* and *"logical"* position which she believes is required for clinical-ethical decision-making. Many participants discussed feeling emotionally invested in patient care and outcomes, and the effects this had on their own mental wellbeing. Beth describes feeling *"wrapped up"* in others' emotions which is *"difficult"* and *"draining"*.
**Torn**	*"Being torn….between what should be done, what can be*	Participants described feeling torn and conflicted often when feeling morally

	done, what you would want for that person as much as what they would want. That feeling as I say of being ripped apart as to what you believe. I suppose that's sometimes the whole point isn't it, it's not supposed to be about necessarily what we believe… that's the confusion of your ethics with the situation." (Liam)	uncertain and caught up in dilemmatic situations. Liam provides a visceral description of how moral distress made him feel, as he describes his belief that moral distress is capable of tearing a person apart. Liam discusses his mistrust of some surgeons and feeling suspicious of their motives when it came to treatment plans. Liam worries that patients receive aggressive treatments because surgeons don't want to damage their mortality figures, rather than because continuing treatment is in the patient's best interests. Perhaps because of this mistrust, Liam seems to believe that when making moral judgments regarding patients, healthcare professionals should set aside their own beliefs to focus on the patient's best interests. Liam's feeling of moral uncertainty also seems to be because of a conflict between his personal and professional beliefs which culminates in feeling torn during moral events.
**Powerlessness**	*"The nurses find it distressing that we've got someone like that here for 3 weeks and you go in and it's futile and you feel like you're being cruel and this gentleman is incontinent and his skin's falling off and it's getting infected and you want to say let's stop, let's stop what we're doing, but the doctors don't and that's what I think I find really, really difficult, you know, when you're at the bedside and you see the nurses and they come in to the office and they say 'I find this really, really hard' and I say yes I know and I agree, I think what we're doing is wrong but we're not in the position... we don't make these decisions and I think*	Some participants accepted the limitations of their role, whereas others described feeling powerless, especially when it came to clinical-ethical decision-making. It may be argued that 'powerlessness' is not an emotion in itself but rather an amalgamation of other emotions, and indeed it seemed to be very often associated with frustration and anger and predominantly occurred during moral conflict and constraint experiences. Nonetheless, it was a feeling that was very often mentioned by participants when recalling their ethical experiences and therefore its inclusion is justified on the basis that it was prevalent in the data. In this quotation, senior nurse Olivia describes her belief that the decision-making hierarchy constrains bedside nurses, causing them to feel powerless because they are obligated to continue providing life-sustaining treatment even if they don't think life-sustaining treatment is morally justified.

	we do try to take on board where they're coming from, especially as senior nurses you have to be there to explain, not justify what the doctors are doing, but you have to back up and say the doctors are doing this, this and this because this is what they think is in the best interests - no we don't agree but we have to work with them." (Olivia)	
**Stress**	*"I was always scared. I had nightmares every night for six months, like I didn't sleep. I would wake up in the night shouting, thinking I was naked, no one was watching my patient, I was naked in the bedside, someone had put my alarms on silent and my patient was arresting. ... just the craziest of nightmares and, you know, until I felt comfortable and safe and confident in my own practice, then I would never question anybody else." (Chloe)*	Many participants discussed feeling stressed because of their experiences, and they discussed symptoms commonly associated with stress such as difficulty sleeping and nightmares. It may be stress that culminates from the various experiences and produces the physical effects of moral distress that have been highlighted in the literature. In this quotation, Chloe discusses the nightmares she experienced when she first started working in ICU because she worried her practice was unsafe and felt she was not adequately supported. The moral issue is not explicit within Chloe's quotation, but arguably organisations are morally responsible for staff wellbeing, and this constitutes a moral event. Many participants described the emotional strain of working in an emotive environment that was then compounded by a lack of staff and resources.
